# The Limits of Comorbidity Indices in TAVI: How Aggregation Dilutes the Prognostic Power of Individual Biomarkers

**DOI:** 10.3390/jcdd13080390

**Published:** 2026-08-14

**Authors:** Nazile Bilgin Doğan, Özdemir Kuzucu

**Affiliations:** Department of Cardiology, Turkish Ministry of Health Izmir Bayraklı City Hospital, Izmir 35540, Turkey; ozdemir.kuzucu@saglik.gov.tr

**Keywords:** transcatheter aortic valve replacement, comorbidity, risk assessment, acute kidney injury, aged, aortic valve stenosis

## Abstract

Objective. Heart-team decisions in transcatheter aortic valve implantation (TAVI) lean on EuroSCORE II, yet the comorbidity burden that often drives the final choice is rarely scored. We tested a specific two-part hypothesis: whether formally quantifying comorbidity burden (via the Charlson Comorbidity Index [CCI] and a modified CCI [mCCI]) recovers prognostic value beyond EuroSCORE II for Valve Academic Research Consortium-3 (VARC-3) outcomes, and whether aggregating comorbidities preserves or dilutes the prognostic signal of individually informative predictors. Methods. In 234 consecutive patients (mean age 76.6 ± 6.1 years) undergoing TAVI for severe aortic stenosis, EuroSCORE II, CCI, and mCCI were computed before the procedure. The primary endpoint was the 30-day VARC-3 composite; secondary endpoints were individual VARC-3 outcomes and one-year mortality. We compared discrimination (AUC, DeLong test) and incremental value with bootstrapping, and analyzed survival by Kaplan–Meier/Cox regression. Results. All three scores discriminated the 30-day composite (63 patients, 26.9%) poorly (AUCs 0.50–0.54), collapsing to chance after correction; neither comorbidity index improved on EuroSCORE II (all *p* > 0.40). EuroSCORE II’s only meaningful signal was stage 3 acute kidney injury (AKI; AUC 0.676), where it significantly exceeded mCCI (*p* = 0.048). Serum albumin and chronic kidney disease (HR 2.97 for one-year mortality) were informative individually but not within an aggregate score. Conclusions. In elderly TAVI, the actionable prognostic information resided in individual markers of organ reserve—serum albumin and chronic kidney disease—that count-based comorbidity aggregation dissipated rather than concentrated. EuroSCORE II retained a focused, mechanistically coherent association with stage 3 AKI, reflecting its renal components. These findings support a shift from disease-counting toward direct measurement of organ reserve and frailty in TAVI risk assessment, and the development of TAVI-specific tools that preserve dominant individual predictors rather than averaging them into a single score.

## 1. Introduction

Transcatheter aortic valve implantation (TAVI) has evolved from a treatment of last resort for inoperable patients into a first-line therapy spanning the entire surgical-risk spectrum, including selected low-risk and, increasingly, elderly candidates. As indications broaden, accurate pre-procedural risk assessment has become central to heart-team decision-making, patient counseling, and institutional benchmarking.

EuroSCORE II and the Society of Thoracic Surgeons (STS) score remain the risk models most frequently discussed in heart-team (cardiology–cardiovascular surgery council) deliberations for severe symptomatic aortic stenosis [1], yet both were derived from surgical populations and weight operative variables that may translate poorly to transcatheter procedures. A consistent body of evidence has documented their miscalibration in contemporary TAVI cohorts, with a tendency toward over-prediction in high-risk and under-prediction in lower-risk strata [2]. Critically, these scores omit a direct, structured measure of cumulative comorbidity burden—a dimension that, in everyday clinical practice, often weighs heavily on the final treatment decision even though it is rarely expressed as a formal score.

This gap between practice and measurement motivated the present study. In our experience, when a heart team converges on a treatment choice, the discussion frequently moves beyond the operative-risk score to the patient’s overall comorbidity load; however, the Charlson Comorbidity Index (CCI)—a validated, freely available summary of comorbidity that is routinely computable from administrative data—almost never enters the formal conversation [3]. The CCI and its modifications incorporating age and nutritional status (here, a modified CCI [mCCI] adding an age-decade weight and a hypoalbuminemia term) have shown prognostic value across diverse cardiovascular settings. This raises the question of whether they could complement, or even outperform, EuroSCORE II in predicting adverse outcomes after TAVI.

We therefore conducted a head-to-head comparison of EuroSCORE II, CCI, and mCCI for the prediction of Valve Academic Research Consortium-3 (VARC-3) outcomes in an elderly TAVI cohort. We advanced two pre-specified hypotheses: first, that comorbidity indices would add prognostic information beyond EuroSCORE II, and second, that aggregating heterogeneous comorbidities into a single ordinal score might dilute the prognostic signal of individually informative predictors (such as serum albumin and renal function) rather than preserve it. The primary aim was to compare the discrimination of the three scores for the 30-day VARC-3 composite endpoint; secondary aims were to evaluate their performance across individual VARC-3 outcomes and one-year mortality, to assess whether either comorbidity index provides incremental value over EuroSCORE II, and to examine—through a discordance analysis and paired individual-versus-aggregate comparisons—whether aggregation obscures dominant individual predictors.

## 2. Methods

### 2.1. Study Design and Population

This was a single-center, retrospective, observational cohort study conducted at the department of cardiology of a tertiary-care city hospital. We screened consecutive patients who underwent TAVI for severe symptomatic aortic stenosis between December 2023 and March 2026. Inclusion criteria were age ≥ 18 years and native or valve-in-valve TAVI with complete baseline risk-score data. Patients with missing baseline EuroSCORE II components were excluded. The study was approved by the institutional clinical research ethics committee (approval no. 2026/399) and conducted in accordance with the Declaration of Helsinki. The study is reported in accordance with the STROBE statement for observational studies.

The final analysis cohort comprised 234 patients. All patients had complete 30-day outcome data. For the one-year mortality endpoint, follow-up was complete in patients whose procedure preceded 15 May 2025 (*n* = 129 had completed ≥12 months); the remaining patients were censored at last contact in the time-to-event analysis.

### 2.2. Risk Scores

EuroSCORE II was calculated for each patient using the official online algorithm [1]. The Charlson Comorbidity Index (CCI) was scored using the original Charlson weighting [3]. A modified CCI (mCCI) was defined a priori as the sum of the CCI, an age-decade weight (0 for <40 years, increasing by 1 point per decade to 5 for ≥81 years), and a 2-point term for hypoalbuminemia (serum albumin < 3.5 g/dL). All three scores were computed from data available before the procedure.

### 2.3. Outcomes

All outcomes were defined according to Valve Academic Research Consortium-3 (VARC-3) consensus criteria [4]. The pre-specified primary endpoint was the 30-day VARC-3 composite (all-cause death, stroke, life-threatening bleeding, major vascular complication, acute kidney injury [AKI] stage ≥ 2, coronary obstruction, or valve-related dysfunction). Secondary 30-day endpoints, analyzed individually, were in-hospital mortality, 30-day all-cause mortality, all stroke, KDIGO AKI stages 1–3, VARC-3 type 1–4 bleeding, major vascular complications, and periprocedural permanent pacemaker implantation. The exploratory long-term endpoint was one-year all-cause mortality.

### 2.4. Statistical Analysis

Continuous variables are presented as mean ± standard deviation (SD) or median (interquartile range, IQR) and compared with the *t*-test or Mann–Whitney U test as appropriate; categorical variables are presented as *n* (%). The normality of the distribution of continuous variables was assessed with the Shapiro–Wilk test, which guided the choice between parametric and non-parametric tests. Collinearity between EuroSCORE II and the comorbidity indices was assessed with the Spearman correlation coefficient.

For each binary outcome, the discriminative performance of EuroSCORE II (log-transformed), CCI, and mCCI was assessed by the area under the receiver-operating-characteristic curve (AUC). Paired AUCs were compared with the DeLong test. Optimal cut-offs were derived by the Youden index, with corresponding sensitivity, specificity, positive and negative predictive values, and accuracy reported with Wilson 95% confidence intervals.

The incremental value of adding a comorbidity index to EuroSCORE II was evaluated by multivariable logistic regression, the likelihood-ratio test for nested models, and category-free net reclassification improvement (NRI) and integrated discrimination improvement (IDI) with bootstrap confidence intervals. To quantify overfitting in this single-center cohort, model discrimination was internally validated using 1000 bootstrap resamples, and optimism-corrected AUCs are reported (Harrell method). Calibration was assessed with the Hosmer–Lemeshow test and the Brier score.

One-year all-cause mortality was analyzed by the Kaplan–Meier method with log-log confidence intervals; groups were compared with the log-rank test. Time-to-event associations were assessed by Cox proportional-hazards regression (all 234 patients, censored at last contact), with hazard ratios (HRs) and 95% confidence intervals; six patients who died on the procedure day were assigned a survival time of 0.5 days. Model discrimination was summarized by Harrell’s C-index. A two-sided *p* < 0.05 was considered statistically significant. Because multiple outcomes were examined, secondary analyses are interpreted as hypothesis-generating. Analyses were performed in Python 3.12 (NumPy, SciPy, scikit-learn).

## 3. Results

### 3.1. Baseline Characteristics

A total of 234 patients undergoing TAVI for severe symptomatic aortic stenosis were analyzed. The mean age was 76.6 ± 6.1 years and 132 patients (56.4%) were women. The mean left ventricular ejection fraction was 54.7 ± 9.4%. The median EuroSCORE II was 2.55% (IQR 1.40–4.88), the median CCI was 5 (IQR 4–6), and the median mCCI was 9 (IQR 7–11). Hypoalbuminemia (<3.5 g/dL) was present in 116 patients (49.6%). The most frequent comorbidities were hypertension (82.9%), diabetes mellitus (53.0%), coronary artery disease (52.1%), atrial fibrillation (17.9%), chronic kidney disease (CKD; 14.5%), and malignancy (13.2%) (Table 1).

Regarding procedural characteristics, transfemoral access was used in 100% of patients, without alternative (transapical/transaxillary/other) access. A balloon-expandable prosthesis was implanted in 57.3% and a self-expanding prosthesis in 42.7%; valve-in-valve procedures accounted for 6% of cases. According to VARC-3 criteria, technical success was achieved in 88% of patients and device success at 30 days in 73.1%.

EuroSCORE II correlated only weakly with the comorbidity indices (Spearman ρ = 0.09 with CCI, *p* = 0.168; ρ = 0.15 with mCCI, *p* = 0.024), whereas CCI and mCCI were strongly correlated (ρ = 0.89), as expected given their shared structure. The weak EuroSCORE–comorbidity correlations indicate that the operative-risk score and the comorbidity indices capture largely non-overlapping patient information.

### 3.2. Thirty-Day Outcomes

The 30-day VARC-3 composite endpoint occurred in 63 patients (26.9%). Individual event rates were 30-day all-cause mortality (27, 11.5%), in-hospital mortality (23, 9.8%), stage 3 AKI (24, 10.3%), VARC-3 type ≥ 3 bleeding (27, 11.5%), major vascular complication (33, 14.1%), all stroke (9, 3.8%), and periprocedural permanent pacemaker implantation (32, 13.7%) (Table 2).

### 3.3. Discrimination of Risk Scores for VARC-3 Outcomes

**Figure 1:**
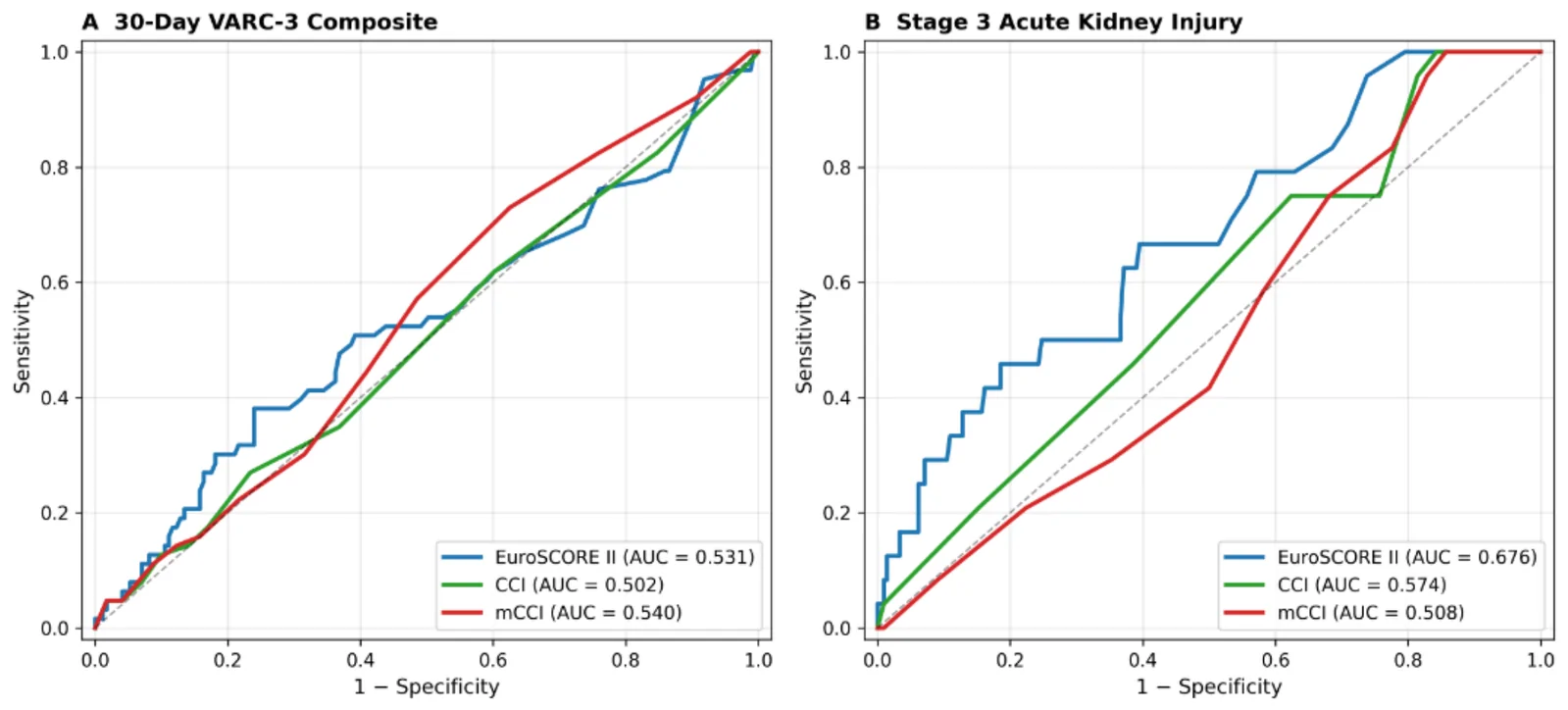
Receiver-operating-characteristic curves for EuroSCORE II, CCI, and mCCI. (**A**) 30-day VARC-3 composite endpoint; all three scores show poor discrimination. (**B**) Stage 3 acute kidney injury; EuroSCORE II shows moderate discrimination, significantly exceeding mCCI (DeLong *p* = 0.048). AUC, area under the curve.

For the primary endpoint (30-day VARC-3 composite), all three scores showed poor discrimination: EuroSCORE II AUC 0.531, CCI AUC 0.502, and mCCI AUC 0.540 (all *p* > 0.30 for the corresponding univariable coefficients). Pairwise DeLong comparisons revealed no significant differences (EuroSCORE II vs. mCCI, *p* = 0.886; CCI vs. mCCI, *p* = 0.087). When both EuroSCORE II and mCCI were entered into a single model, neither retained significance (EuroSCORE II *p* = 0.372; mCCI *p* = 0.444), and the likelihood-ratio test confirmed that mCCI added no incremental information over EuroSCORE II (*p* = 0.447). Reclassification metrics were negligible (NRI +0.030; IDI +0.0022) (Figure 1A, Table 3).

Across the remaining VARC-3 outcomes, neither comorbidity index added significant predictive value to EuroSCORE II on likelihood-ratio testing (all *p* > 0.60). The single exception to the overall pattern of poor discrimination was stage 3 AKI.

### 3.4. EuroSCORE II and Stage 3 Acute Kidney Injury

EuroSCORE II discriminated stage 3 AKI with moderate accuracy (AUC 0.676, *p* = 0.002), significantly outperforming mCCI (AUC 0.508; DeLong *p* = 0.048) and numerically exceeding CCI (AUC 0.574; DeLong CCI vs. mCCI *p* = 0.057). At the Youden-optimal cut-off (EuroSCORE II ≥ 5.70%), sensitivity was 45.8% and specificity 81.4% for stage 3 AKI. In a model containing both scores, EuroSCORE II remained significant (*p* = 0.002) while mCCI did not (*p* = 0.242). This renal-specific signal is consistent with the inclusion of creatinine within the EuroSCORE II algorithm (Figure 1B).

Notably, although the composite comorbidity index (mCCI) failed to predict AKI, serum albumin analyzed as an individual variable was significantly associated with stage 3 AKI: 14.7% in hypoalbuminemic versus 5.9% in normoalbuminemic patients (OR 2.72, *p* = 0.032). A parallel pattern was observed for CKD, which as a single comorbidity was the strongest individual predictor of adverse outcomes. These findings suggest that individually informative biomarkers lose their prognostic signal when aggregated into a composite comorbidity score.

### 3.5. Internal Validation

To address overfitting in this single-center cohort, optimism-corrected AUCs were derived from 1000 bootstrap resamples. For the VARC-3 composite, all models converged toward chance after correction (EuroSCORE II 0.506, CCI 0.473, mCCI 0.522, EuroSCORE II + mCCI 0.517). In contrast, the EuroSCORE II–AKI association was essentially unaffected by optimism correction (apparent and corrected AUC both 0.676), confirming the robustness of this single positive finding.

### 3.6. Discordance Analysis

Because heart-team discussions often center on patients who appear low-risk by operative score yet carry a heavy comorbidity burden, we performed a discordance analysis. Using clinically anchored cut-offs (EuroSCORE II 4% and the median mCCI of 9), patients were classified into four groups (Table 4). The clinically pivotal group comprised 76 patients (32.5%) judged low-risk by EuroSCORE II (<4%) but high-risk by mCCI (≥9)—the “hidden high-comorbidity” phenotype. Compared with the 84 concordantly low-risk patients (EuroSCORE II < 4%, mCCI < 9), this group did not have significantly worse outcomes for any endpoint: VARC-3 composite 26.3% versus 22.6% (*p* = 0.713), 30-day mortality 11.8% versus 7.1% (*p* = 0.417), and one-year mortality 17.1% versus 11.9% (*p* = 0.375). Notably, no stage 3 AKI events occurred in either low-EuroSCORE subgroup.

Across the full four-group table, the gradient in adverse outcomes tracked the EuroSCORE II axis rather than the mCCI axis: 30-day and one-year mortality were highest in the two high-EuroSCORE groups (16.1–16.3% and 25.6–29.0%, respectively) regardless of mCCI status, whereas reclassifying a low-EuroSCORE patient as high-risk by mCCI did not identify a meaningfully higher-risk subset. Thus, even in the specific subgroup where the comorbidity index most strongly contradicted the operative score, mCCI did not provide clinically useful prognostic separation.

### 3.7. One-Year Mortality

Over a median follow-up of 402 days, 44 deaths occurred. Kaplan–Meier estimated 30-day and 1-year survival were 90.2% (95% CI 85.6–93.4) and 82.3% (95% CI 76.8–86.7), respectively. In univariable Cox regression, neither EuroSCORE II (HR 1.25 per doubling, 95% CI 0.99–1.58, *p* = 0.062), CCI (HR 0.98, *p* = 0.698), nor mCCI (HR 1.02, *p* = 0.687) was significantly associated with all-cause mortality. After multivariable adjustment, none of the scores reached significance, and model discrimination was poor (C-index 0.58).

**Figure 2:**
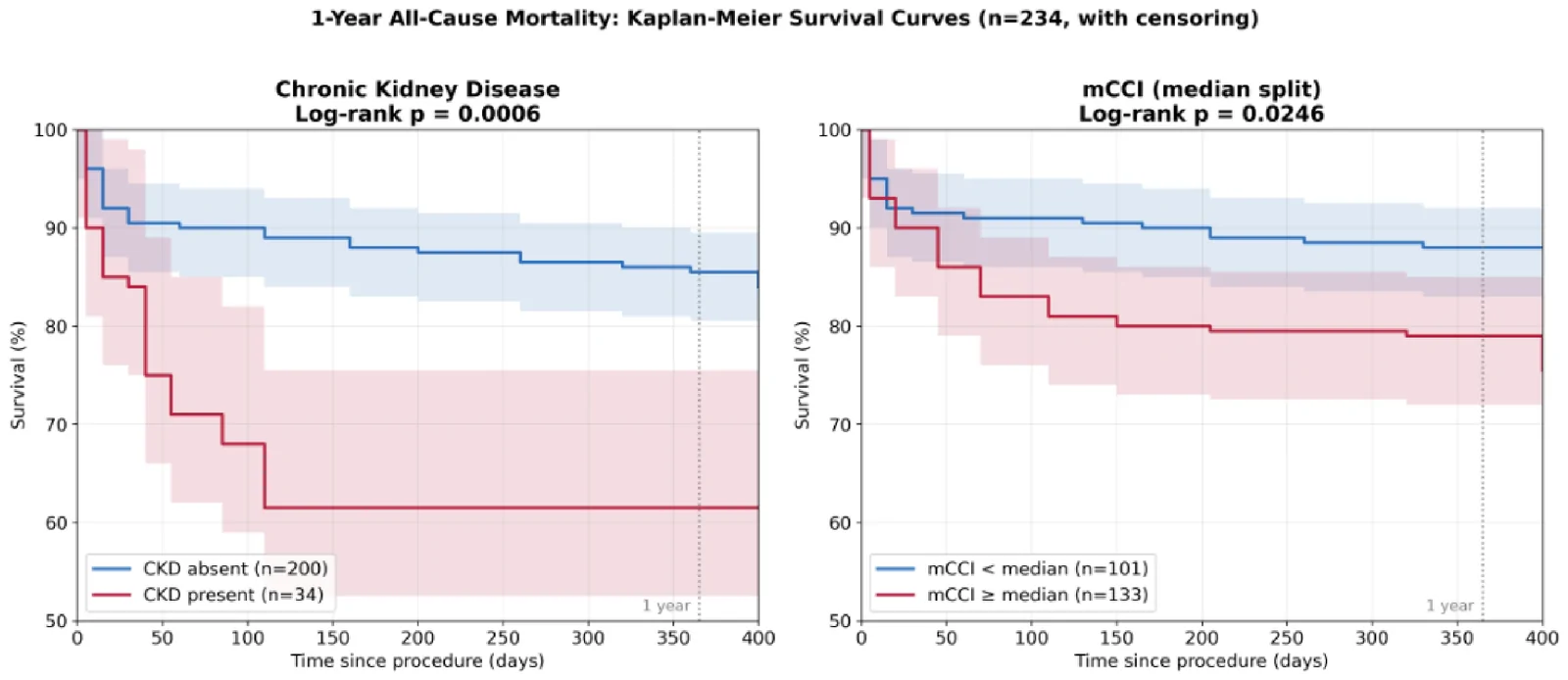
One-year survival (Kaplan–Meier). All-cause mortality stratified by chronic kidney disease (**left**) and by mCCI median split (**right**), with log-rank *p* values. Shaded areas denote 95% confidence intervals.

By contrast, CKD as a single variable was strongly associated with mortality (HR 2.97, 95% CI 1.55–5.69, *p* = 0.001; C-index 0.76), with a log-rank *p* = 0.0006 between groups; 1-year survival was approximately 62% in patients with CKD versus 85% in those without (Figure 2).

Given that continuous scores failed to predict mortality, we additionally examined whether dichotomizing the indices recovered any prognostic signal. A median split of mCCI (≥9 versus <9) did separate the survival curves (log-rank *p* = 0.025; Figure 2), whereas neither EuroSCORE II nor CCI median splits reached significance. This is the only configuration in which a comorbidity index showed a mortality signal. However, this separation should be interpreted cautiously: it was driven largely by the age-decade component of the mCCI rather than by comorbidity per se, since the comorbidity-only CCI—which lacks the age term—showed no such separation. In other words, what the dichotomized mCCI captured was predominantly chronological age, not an incremental contribution of comorbidity counting.

## 4. Discussion

In this single-center cohort of elderly patients undergoing TAVI, we identified three findings with direct implications for pre-procedural risk assessment. First, the actionable prognostic information lay in individually measured markers of organ reserve: serum albumin was associated with stage 3 acute kidney injury (AKI), and chronic kidney disease was the dominant predictor of one-year mortality (HR 2.97)—yet both lost their signal once embedded within a composite comorbidity score. Second, EuroSCORE II retained a focused, bootstrap-stable association with stage 3 AKI (AUC 0.676), significantly outperforming the modified Charlson index; this renal-specific signal is most plausibly attributable to the creatinine term embedded within the EuroSCORE II algorithm. Third, and unifying these observations, formal aggregation of heterogeneous comorbidities dissipated rather than concentrated the prognostic signal of the few individual variables that genuinely drive risk—providing a mechanistic explanation for why neither EuroSCORE II nor the comorbidity indices discriminated the 30-day VARC-3 composite or one-year mortality after bootstrap correction.

### 4.1. The Practice–Measurement Gap

Our starting point was a familiar clinical observation: in heart-team deliberations, the final treatment decision often hinges on the patient’s overall comorbidity burden, yet this burden is seldom captured by a formal score. We tested whether translating that clinical intuition into a structured index (CCI/mCCI) would improve outcome prediction. It did not. This is, in itself, an informative result: the prognostic weight that clinicians intuitively assign to comorbidity was not recovered by either comorbidity index in our cohort. One interpretation is that the clinical gestalt integrates information—frailty, functional reserve, and the trajectory of individual organ dysfunction—that a count-based comorbidity score cannot represent. The negative finding does not invalidate the clinical instinct; rather, it highlights that currently available aggregate scores are inadequate surrogates for it.

### 4.2. Aggregation Dilutes Individually Informative Predictors

A recurring pattern in our data offers a mechanistic explanation for the failure of the comorbidity indices. Serum albumin, analyzed as a single variable, was significantly associated with stage 3 AKI (OR 2.72), yet incorporating it into the mCCI did not improve—and numerically reduced—discrimination. This accords with prior work linking low preprocedural albumin to post-TAVI AKI and mortality [5,6]. Consistent with this, composite immunonutritional indices that retain albumin as a component (e.g., the C-reactive protein–albumin–lymphocyte [CALLY] index) predict early mortality after TAVI [7], reinforcing that nutritional status carries prognostic weight in this setting. Likewise, chronic kidney disease as a single comorbidity was the strongest individual predictor of one-year mortality (HR 2.97), yet this signal was not detectable through the CCI, in which renal disease is one weighted item among many. For TAVI specifically, where renal and nutritional status are dominant determinants of early complications, a composite that gives equal weight to less relevant comorbidities may obscure rather than clarify risk.

### 4.3. EuroSCORE II and Acute Kidney Injury

**Figure 3:**
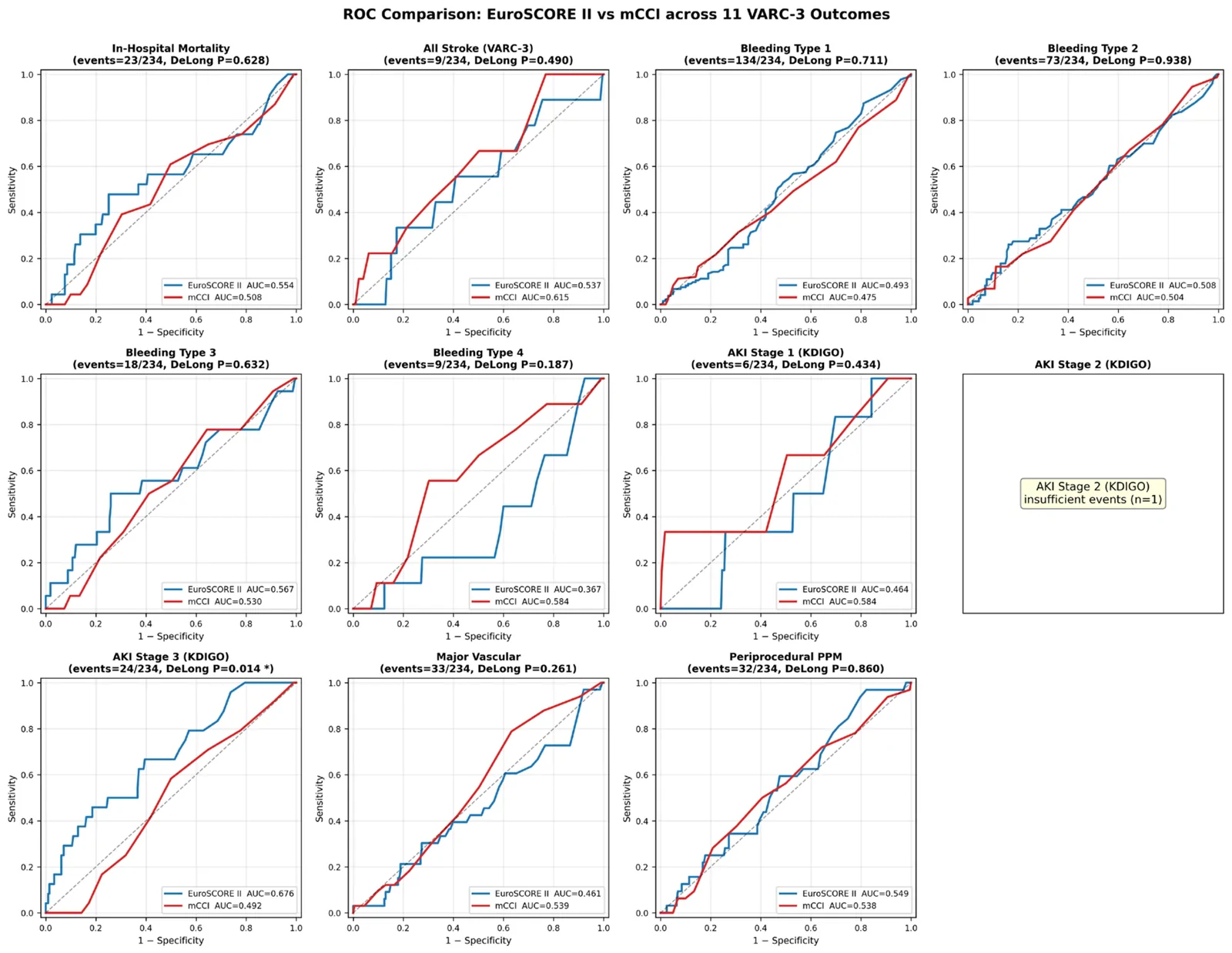
ROC comparison of EuroSCORE II versus mCCI across 11 individual VARC-3 outcomes. Each panel shows receiver-operating-characteristic curves for EuroSCORE II and mCCI for one VARC-3 outcome, with the number of events and the DeLong *p* value for the AUC comparison. The only statistically significant difference favored EuroSCORE II for stage 3 acute kidney injury (*p* = 0.014 *). AKI stage 2 had insufficient events for analysis. AUC, area under the curve; PPM, permanent pacemaker.

The sole domain in which EuroSCORE II retained meaningful discrimination was stage 3 AKI (AUC 0.676), where it significantly exceeded mCCI (Figure 3). This is consistent with the score’s incorporation of renal function (creatinine/creatinine clearance) and with the established role of baseline renal impairment as a precursor of post-procedural AKI. The finding should be interpreted cautiously: because creatinine is a EuroSCORE II input and renal impairment a substrate for AKI, this association is partly structural rather than a demonstration of genuine added clinical value. Nevertheless, it indicates that EuroSCORE II may retain a focused, renal-oriented predictive role even where its global discrimination for composite outcomes is poor.

### 4.4. Comparison with Prior Literature

Our results align with reports questioning the calibration and discrimination of surgical risk scores in contemporary TAVI populations [2,8,9], and extend them by directly contrasting EuroSCORE II with comorbidity indices in an elderly cohort. Frailty instruments (e.g., the Essential Frailty Toolset, gait speed, Katz index) have been proposed as adjuncts and may capture the reserve-related information that neither EuroSCORE II nor the CCI reflects [10]; our data indirectly support this view, since a count-based comorbidity score did not bridge the gap. A recent multidimensional comparison in TAVI similarly found that conventional surgical risk scores insufficiently captured functional and cognitive vulnerability relative to dedicated frailty and biological scores [11]. The weak EuroSCORE II–comorbidity correlation we observed (ρ ≤ 0.15) confirms that these tools measure largely distinct domains, yet neither domain alone proved sufficient for robust outcome prediction. Our renal-specific EuroSCORE II signal is likewise consistent with the recognized prognostic weight of acute kidney injury in TAVI, whose temporal association with mortality is strongest in the early post-procedural period [12].

### 4.5. Comorbidity Count Is Not Frailty

The central interpretive question raised by our findings is why a structured comorbidity index, which formalizes the very burden that clinicians weigh so heavily, failed to predict outcomes. We propose that the answer lies in a distinction well established in geriatric medicine: comorbidity, frailty, and disability are related but distinct clinical entities [13]. Counting diseases is not the same as measuring physiological reserve. The CCI and mCCI quantify how many—and which—diagnoses a patient carries (diabetes, hypertension, prior myocardial infarction, and so on), but they are blind to whether that patient walks into the clinic unaided or arrives in a wheelchair. Two patients with an identical mCCI may have entirely different functional trajectories after TAVI, and it is the latter—gait speed, muscle strength, nutritional and cognitive status—that determines recovery [10]. This is precisely the domain captured by dedicated frailty instruments and not by disease enumeration.

Our own data illustrate the point from the opposite direction. The variables that did carry prognostic signal in this cohort were not comorbidity counts but direct markers of organ reserve and physiological depletion—serum albumin as an index of nutritional status and frailty, and renal function as an index of physiological reserve—precisely the reserve-measuring domain that disease enumeration cannot represent. In this light, the failure of the mCCI is not a failure of the comorbidity concept but a reflection of where prognostically decisive information in elderly TAVI patients actually resides. Our findings therefore argue that risk assessment in this population should move away from counting comorbidities and toward directly measuring organ reserve (renal function, nutritional status) and frailty. We stress, however, that this interpretation is inferential: because no frailty instrument was administered, the superiority of physiological reserve over comorbidity burden rests on reserve-related surrogates rather than on a direct frailty measurement, and warrants confirmation in cohorts that prospectively apply validated frailty tools.

### 4.6. Clinical Implications

For the elderly TAVI population, our findings counsel against substituting or supplementing EuroSCORE II with a generic comorbidity index in the expectation of improved outcome prediction. Where renal risk is the specific concern, EuroSCORE II—or, more directly, baseline renal function and serum albumin as individual variables—may be more informative than an aggregate comorbidity score. In practical terms, we do not propose serum albumin and renal function as standalone replacements for existing scores. Rather, together with a frailty measure, they are candidate components of a future integrated, TAVI-specific model in which dominant predictors retain their individual weight instead of being averaged into a single count. Until such a model is developed and validated, Heart Teams should treat low serum albumin or impaired renal function as explicit warning signals alongside EuroSCORE II, rather than adopting a formal comorbidity index in their place. Because these observations derive from a single-center cohort, they remain hypothesis-generating and require external, multicenter validation before they can justify any change in routine clinical risk assessment.

## 5. Study Limitations

This study has several limitations. First, it is a single-center, retrospective analysis of modest size (*n* = 234), which limits generalizability and statistical power for less frequent outcomes such as stroke. Accordingly, secondary analyses are hypothesis-generating, and we applied bootstrap internal validation to temper optimism. In addition, our comparison was deliberately restricted to EuroSCORE II and Charlson-based indices and did not incorporate TAVI-specific prediction models (such as the TAVI2-SCORe, FRANCE-2, or OBSERVANT scores) or dedicated frailty instruments. These tools were not systematically recorded in our institutional database during the study period and could not be reconstructed retrospectively with adequate fidelity. Conceptually, these tools also differ from generic comorbidity indices in that they were derived and calibrated specifically for the transcatheter population and, in the case of frailty instruments, capture the functional, nutritional, and cognitive reserve that disease-counting scores omit—precisely the dimensions our findings identify as prognostically decisive. Although this focused comparison reflects the scores most often debated in our Heart Team, the absence of TAVI-specific and frailty-oriented models limits the comparative scope of our analysis. Future studies should benchmark comorbidity indices against these contemporary tools. Second, one-year outcomes were available for only part of the cohort; we addressed this with time-to-event analysis and censoring, but longer and more complete follow-up is needed. Third, we did not include a dedicated frailty assessment, which may capture prognostic information beyond both EuroSCORE II and the comorbidity indices. This limitation is especially consequential here, because the central interpretation of our results, that physiological reserve and frailty are more informative than comorbidity burden, cannot be tested directly without a frailty measure. Our inference on this point rests on reserve-related surrogates (serum albumin, renal function) rather than on a validated frailty instrument, and should therefore be regarded as provisional. Fourth, as a study confined to patients who actually underwent TAVI, it cannot address the selection decision made by the heart team, and a degree of selection bias is inherent. Finally, external, multicenter validation is required before our conclusions can be generalized to other populations and practice settings.

## 6. Conclusions

In elderly patients undergoing TAVI, the prognostically decisive information resided in individual markers of organ reserve rather than in comorbidity counts. For daily heart-team practice, our findings suggest a concrete refinement: prioritize direct measurement of serum albumin and renal function alongside EuroSCORE II, rather than adding a composite comorbidity index that averages away their signal. EuroSCORE II retained a focused, internally validated association with stage 3 AKI, likely reflecting its renal components, but offered no useful discrimination for composite outcomes or mortality. For future research, these findings support the development and validation of TAVI-specific, multidimensional risk tools that preserve the predictive strength of dominant individual factors—renal function, nutritional status, and frailty—rather than diluting them within an aggregate score. Because these findings derive from a single-center, retrospective cohort, they should be regarded as hypothesis-generating and require prospective, multicenter external validation before they can support any change in routine clinical risk assessment.

## Figures and Tables

**Table 1:** Baseline characteristics of the study cohort.

Variable	All Patients (*n* = 234)
Age, years—mean ± SD	76.6 ± 6.1
Female—*n* (%)	132 (56.4)
LVEF, %—mean ± SD	54.7 ± 9.4
Serum albumin, g/dL—mean ± SD	3.40 ± 0.34
Hypoalbuminemia < 3.5 g/dL—*n* (%)	116 (49.6)
EuroSCORE II, %—median (IQR)	2.55 (1.40–4.88)
CCI—median (IQR)	5 (4–6)
mCCI—median (IQR)	9 (7–11)
Diabetes mellitus—*n* (%)	124 (53.0)
Hypertension—*n* (%)	194 (82.9)
Coronary artery disease—*n* (%)	122 (52.1)
Chronic kidney disease—*n* (%)	34 (14.5)
COPD—*n* (%)	29 (12.4)
Atrial fibrillation—*n* (%)	42 (17.9)
Malignancy—*n* (%)	31 (13.2)

Data are mean ± SD, median (IQR), or *n* (%). CCI, Charlson Comorbidity Index; COPD, chronic obstructive pulmonary disease; IQR, interquartile range; LVEF, left ventricular ejection fraction; mCCI, modified Charlson Comorbidity Index; SD, standard deviation.

**Table 2:** Thirty-day VARC-3 outcomes.

30-Day VARC-3 Outcome	*n* (%)
VARC-3 composite	63 (26.9)
30-day all-cause mortality	27 (11.5)
In-hospital mortality	23 (9.8)
Stage 3 acute kidney injury	24 (10.3)
VARC-3 type ≥ 3 bleeding	27 (11.5)
Major vascular complication	33 (14.1)
All stroke	9 (3.8)
Periprocedural permanent pacemaker	32 (13.7)

VARC-3, Valve Academic Research Consortium-3.

**Table 3:** Discrimination (AUC) of EuroSCORE II, CCI, and mCCI for selected outcomes.

Outcome	EuroSCORE II	CCI	mCCI
VARC-3 composite	0.531	0.502	0.540
Stage 3 AKI	0.676	0.574	0.508
30-day mortality	0.552	0.502	0.525

AKI, acute kidney injury; AUC, area under the receiver-operating-characteristic curve; CCI, Charlson Comorbidity Index; mCCI, modified Charlson Comorbidity Index. For the VARC-3 composite, optimism-corrected AUCs after 1000 bootstrap resamples were 0.506, 0.473, and 0.522 for EuroSCORE II, CCI, and mCCI, respectively; the EuroSCORE II AUC for stage 3 AKI was unchanged after correction (0.676).

**Table 4:** Discordance analysis: 30-day and one-year outcomes by EuroSCORE II/mCCI concordance group.

Concordance Group	*n*	VARC-3, *n* (%)	30-Day Mortality, *n* (%)	1-Year Mortality, *n* (%)
Concordant low (ES < 4, mCCI < 9)	84	19 (22.6)	6 (7.1)	10 (11.9)
Discordant: low ES/high mCCI	76	20 (26.3)	9 (11.8)	13 (17.1)
Discordant: high ES/low mCCI	31	8 (25.8)	5 (16.1)	9 (29.0)
Concordant high (ES ≥ 4, mCCI ≥ 9)	43	16 (37.2)	7 (16.3)	11 (25.6)

Groups defined by a EuroSCORE II cut-off of 4% and the median mCCI of 9. The “discordant: low ES/high mCCI” group represents patients judged low-risk by EuroSCORE II but high-risk by mCCI. ES, EuroSCORE II; mCCI, modified Charlson Comorbidity Index; VARC-3, Valve Academic Research Consortium-3.

## Data Availability

The data presented in this study are available on reasonable request from the corresponding author. The data are not publicly available owing to privacy and ethical restrictions.
